# Searching for Social Media Addiction: A Content Analysis of Top Websites Found through Online Search Engines

**DOI:** 10.3390/ijerph181910077

**Published:** 2021-09-25

**Authors:** Alexis M. McCarroll, Bree E. Holtz, Dar Meshi

**Affiliations:** 1 Department of Communication and Media, University of Michigan, Ann Arbor, MI 48109, USA; mcalexis@umich.edu; 2 Department of Advertising and Public Relations, Michigan State University, East Lansing, MI 48824, USA; bholtz@msu.edu

**Keywords:** health literacy, health information, content analysis, websites, social media addiction, problematic social media use

## Abstract

Disordered social media use, often referred to as “social media addiction”, has not been officially recognized by medical bodies such as the American Psychiatric Association or the World Health Organization. However, websites still present information to laypeople on how to treat and manage social media addiction, which can pose the risk of spreading low quality or incorrect information. As such, we aimed to assess how the most popular social media addiction websites present information across multiple metrics. We conducted an in-depth online search to identify the top social media addiction websites in November 2019 (N = 23). Websites were separated into four distinct classifications: (1) treatment/therapy/medical; (2) informational; (3) news article; and (4) blog/essay. Based on previous website analysis research, three trained coders evaluated these websites on six metrics: (1) design; (2) credibility; (3) accessibility; (4) literacy; (5) engagement; and (6) social media addiction content. Design features were the top-rated metric across all websites, followed by credibility. Websites scored the lowest for the engagement and social media addiction content metrics. Across website classifications, scores for social media addiction content varied greatly, with blog/essay websites ranking the lowest and informational websites ranking the highest. Our findings provide necessary information for both patients and healthcare providers, apprising these individuals and the field about the current online health information landscape for disordered social media use.

## 1. Introduction

The internet is an integrated part of the daily lives of millions of people, with 90% of Americans adults connected online [1]. As internet use becomes more widespread and accessible, more people than ever turn to it as their initial source for health information [2,3,4]. Unlike traditional methods of obtaining health information from reliable sources (e.g., healthcare providers and medical professionals), online health sources pose the risk of spreading low quality or false information that could contribute to worse health outcomes [5]. Additionally, individuals seeking health information online may not have the necessary skills or literacy to assess content, which can lead to individuals further believing misinformation [4]. For these reasons, it can be challenging for individuals to find information that is relevant, credible, and understandable in order to enact healthy behaviors [6].

With this in mind, we decided to examine the online information available regarding the excessive, maladaptive use of social media platforms. This disordered social media use is often referred to as “social media addiction” or “social networking addiction”, though there is still debate among scholars about whether maladaptive social media use should be termed an “addiction” [7,8]. Individuals who excessively use social media display similar, hallmark symptoms that are present with substance use and behavioral addictive disorders, such as salience, tolerance, mood modification, conflict, withdrawal, and relapse [7]. The effects of this problematic social media use can be significant—it has been linked to job loss, poor academic performance, psychological distress, impaired daily functioning, and poor mental health [9,10,11]. Excessive social media use has garnered so much attention in the US that members of Congress have introduced legislation in an attempt to reduce Americans’ use of social media [12].

More importantly, however, disordered social media use is not formally recognized by the American Psychiatric Association’s (APA) Diagnostic and Statistical Manual of Mental Disorders, Fifth Edition (DSM-V) or the World Health Organization’s (WHO) International Classification of Diseases, 11th version (ICD-11) [13,14]. Therefore, there are no established criteria to diagnose disordered social media use. Because of this, we suspect that information about the symptoms and diagnosis of disordered social media use may vary widely online. Particularly, online platforms such as blogs or opinion pieces may pose a greater risk of misinformation than articles that have undergone rigorous peer-review by experts within the field. While there are existing analyses on web-based information about substance abuse disorders [15,16,17], a formal evaluation of available online information about disordered social media use has not yet been conducted. Such an evaluation is critical given the growing concern over disordered social media use in recent years [18,19,20]. Importantly, this information may be sought out by: (1) individuals who think they may have disordered social media use, (2) family and friends of individuals at risk for disordered social media use, and (3) healthcare providers and educators who would like to equip patients and their family members with reliable online sources.

Therefore, our overall aim was to evaluate available online information about disordered social media use. In addition to assessing content concerning social media addiction, we assessed websites using other quality indicators, such as design, credibility, accessibility, literacy, and engagement (described in the Materials and Methods section). We used a descriptive coding tool that has been used for previous content analyses regarding online health information [21,22]. Rather than testing formal hypotheses, we used this framework to conduct a descriptive study about the existing landscape of social media addiction websites. This approach is characteristic of qualitative research, where the results can help generate hypotheses rather than test them [23,24].

## 2. Materials and Methods

### 2.1. Sample Selection

We conducted a broad online search consisting of 38 separate queries to identify the top social media addiction websites. These queries were conducted in November 2019 from a computer located in the United States. First, we created a list of 38 probable search terms related to social media addiction (see Appendix A for all search terms used). Of note, although using the term “addiction” is still debated in the academic field, this term is used quite often in modern society, and hence, people are likely to conduct online searches for health information with this term. This is supported by data from Google Trends, which reveal that the term “social media addiction” is more frequently searched than related academic and clinical terms such as “problematic social media use” or “social media disorder” [25]. Therefore, we primarily used the term “addiction” to emulate the general online search results that people would obtain, although several of our searches did include terms such as “problematic”, “disorder”, and “excessive”. Next, while our browser was in privacy mode, we entered these terms into three of the most commonly used online search engines: Google, Bing, and Yahoo [26]. For each search engine, we recorded the first page (10 websites) of search results for each term. We did not record websites returned after the first page of results because research has demonstrated that the majority of people rarely continue past the first page [27,28]. In order to be included in the analysis, a website had to appear in the results for six or more of the search terms across all three search engines. This selection process was done to ensure that the websites included in the assessment are seen by the majority of people searching for information related to social media addiction. This resulted in a total of 23 websites for analysis (Table 1).

### 2.2. Coding Instrument

We used a descriptive coding instrument based on two previous studies of telehealth and diabetes websites [21,22]. Social media addiction websites were evaluated on the following six categories: design, credibility, accessibility, literacy, engagement, and social media addiction content. Each of the categories’ items were developed based on previous literature and guidelines for building websites. Therefore, this descriptive coding instrument has face validity for this study, as well as the previous studies. For exact scoring criteria for each of these factors, please see Table 2, Table 3, Table 4, Table 5, Table 6 and Table 7. Briefly, design was evaluated based on the websites’ use of home page links, search function, logos, crumb trails, graphics, layout, and other aspects of web page design. Credibility criteria included the website’s mission statement, terms of service, current copyright year, and whether or not an author or editor was listed. Criteria for accessibility included whether or not font size could be enlarged, web pages were mobile-friendly, content was available in multiple languages, and images have alternative text. Literacy was assessed by reading level using a readability analyzer online (from https://datayze.com/readability-analyzer.php, accessed on 27 December 2019). The websites’ use of graphs, charts, tables, as well as fractions, decimals, percentages, and descriptive statistical terms (i.e., words such as mean, median, and mode) were also used to assess numerical literacy. Criteria for engagement was used to measure the ways users could interact with the website, including sharing content on social media profiles, signing up for email alerts, contacting the organization, or becoming a member. These were only noted in the evaluation if the organization included links to them on their website. All websites were assessed for social media addiction content, including definitions, symptoms, diagnosis, treatment, strategies to reduce social media use, and materials for healthcare providers and family members of an individual with social media addiction.

### 2.3. Coding Procedures and Analysis

The social media addiction websites were coded by three trained coders between December 2019 and May 2020. To establish inter-coder reliability, coders evaluated the same six websites independently over three rounds. After each round of coding, the research team met to discuss disagreements until percent agreement for each of the six categories was greater than 80%. After this was met, the coders assessed the remaining websites individually. Most of the questions in the assessment were answered using “yes”, “no”, and “not applicable”. The responses were entered into a spreadsheet to calculate frequencies for the six categories (design, credibility, accessibility, literacy, engagement, and social media addiction content).

### 2.4. Classification of Websites

We classified all 23 social media addiction websites into four different types of websites: (1) treatment/therapy/medical; (2) informational; (3) news article; and (4) blog/essay. We defined the treatment/therapy/medical classification to include organizations that can provide treatment or a link to treatment (such as hospitals). This classification included six websites. The informational classification, which included seven websites, was defined to include university websites, government websites, journal articles, and Wikipedia. We defined the news article classification to include the four webpages that came from online newspapers, such as The Washington Post. We defined the last classification, blog/essay, to include the six websites ran by an individual or small group that published informal posts. Our analysis compared each of these four website classifications across the six metrics of the coding instrument.

## 3. Results

### 3.1. Design

We evaluated each websites’ design with 25 items (Table 2). All websites met 17 of the 25 criteria, including the ability to access the websites on multiple browsers, and the consistent use of logos, page headers, graphics, colors, font size, and style. All websites made use of white space and displayed the article name on the top window (tab) bar, with all but one website displaying a logo on the top window bar. Nineteen websites provided a search function and clearly displayed the search results, however only seven of these indicated the scope of the search (i.e., just the website or all of the internet). Seven of the 23 websites included their most important information “above the fold,” meaning that no scrolling was necessary.

Across the four website classifications, design scores were similar. Blog/essay websites included 76% to 88% (mean 83%) of the 25 items; news articles had scores that ranged from 80% to 92% (mean 85%); treatment/therapy/medical websites had scores that ranged from 76% to 92% (mean 86%); informational websites scored the highest for the design assessment, with scores that ranged from 80% to 88% (mean 87%) of the design features that were evaluated. These findings indicate that design features are typically consistent across all websites.

### 3.2. Credibility

We evaluated each websites’ credibility with 12 items (Table 3). No website met all 12 of the credibility items. Fourteen websites noted an author, editor, and/or reviewer. Fifteen websites explicitly disclosed their purpose, goal, and/or mission. Of the eight websites that did not explicitly state this information, seven of them provided information about the organization’s purpose. Twenty websites provided copyright information, with nineteen of those websites including copyright for the current year (2020). Eighteen websites included a privacy statement, and all of these privacy statements included a mention of cookies. Fifteen websites had a “terms of use” or “terms of service” section. Twelve of these websites included references to outside sources for their information at least once.

Only three of the websites indicated that their content was reviewed by “experts.”

Treatment/therapy/medical websites scored the lowest for credibility items, with scores that ranged from 33% to 83% (mean 56%) of the 12 items. Five of the six treatment/therapy/medical websites did not list an author, editor, or reviewer, nor did they indicate that their content was reviewed by experts or present reference for their informational content at least once. Additionally, five of these websites did not mention the use of cookies in their privacy disclaimer or the creation date of the webpage, and four did not include the date of the last webpage update. Blog/essay websites had scores that ranged from 42% to 83% (mean 60%). No blog/essay website included the date of the last update or an indication that the content was reviewed by experts. Informational websites had scores that ranged from 58% to 92% (mean 67%). The NewLifeOutlook website had the highest score; it included 11 out of 12 credibility features. News articles scored the highest for the credibility items and had scores from 67% to 83% (mean 77%). No news article website indicated that they were reviewed by experts, but they met the other credibility items.

### 3.3. Accessibility

We evaluated each websites’ accessibility using 15 items (Table 4). None of the websites allowed users to adjust the font size or listen to written material. All websites provided sufficient color contrast. Across all websites, users were able to navigate using their keyboard, and 17 of those websites used a focus indicator to show users where they are on the webpage. Only three websites allowed users to view content in another language. Four websites had videos, but only one of those websites provided closed captioning on their videos. Twenty websites included pictures, but only four of the sites included text that explained those images.

Accessibility scores were low across all website classifications. Blog/essay websites had scores that ranged from 33% to 47% (mean 39%) of the 15 items; informational websites had scores from 33% to 60% (mean 45%); treatment/therapy/medical websites had scores from 33% to 67% (mean 48%); and news articles scored the highest for accessibility, with scores ranging from 47% to 60% (mean 57%). These scores indicate that current social media addiction websites may lack several accessibility features, which may make it difficult for some users to retrieve information.

### 3.4. Literacy

We assessed each websites’ literacy using 15 items (Table 5). Numbers were used on 22 of the websites, with 15 websites using percentages, 11 websites using ranges, nine websites using decimals, seven websites using fractions, and seven websites using descriptive statistical terms such as mean, average, and median. Twenty-two websites used temporal terms such as minutes, hours, and days. Only two websites used graphs, charts, or tables to display data. Eighteen of the websites, allowed users to easily print information, and ten of the websites allowed users to easily send information to somebody else. Nine websites included advertisements—seven of which explicitly labeled them, and five of which had advertisements that moved, flashed, or changed.

Regarding readability, the number of sentences on the websites ranged from 28 to 292, with an average of 92.1 sentences per website. The number of words per sentence ranged from 12.1 to 23.9, with an average of 18.5 words per sentence. The number of characters per word ranged from 4.6 to 5.8, with an average of 5.1 characters per word. The websites had an average Flesch Reading Ease score of 40, an average Flesch–Kincaid Grade Level of 12.5 (12th grade), and an average Simple Measure of Gobbledygook (SMOG) grade of 15.1.

Treatment/therapy/medical websites had readability scores that ranged from 20% to 60% (mean 41%) of the 15 items. No website in this classification had advertisements, nor could an individual easily send information to someone else. Informational websites had scores from 33% to 80% (mean 54%). News article websites scored from 46% to 73% (mean 58%). Blog/essay websites scored the highest on average, with scores from 33% to 87% (mean 60%). Treatment/therapy/medical websites had the lowest average reading level, with an average Flesch–Kincaid Grade Level of 11.3 and an average SMOG grade of 14.1. Informational websites had the highest average reading level, with an average Flesch–Kincaid Grade Level of 14.2 and an average SMOG grade of 16.1. Blog/essay websites had an average Flesch–Kincaid Grade Level of 11.4 and an average SMOG grade of 14.5, and News article websites had a Flesch–Kincaid Grade Level of 12.9 and an average SMOG grade of 15.7.

### 3.5. Engagement

We evaluated website engagement using 16 items (Table 6). Social media presence of all websites was assessed for Facebook, Twitter, YouTube, Pinterest, and Instagram. While twenty websites included a link to their Facebook profiles, only eight of them had verified Facebook accounts. Fourteen websites had a presence on Twitter, with only four being verified. Eleven websites had a presence on Instagram, but only two of them had verified accounts. Ten websites had their own YouTube channels, but only two of those channels were verified. Seven websites had a presence on Pinterest, and only one of those websites was verified. Twenty websites included a “Contact Us” section. One website explicitly asked individuals to donate money, two websites asked individuals to become a member or get actively involved with their organization, and no websites asked individuals to volunteer.

Across the classifications, scores for the engagement items were low. Informational websites scored the lowest, with scores from 6% to 44% (mean 21%) of the 16 items. Three websites in this classification had a “Contact Us” section but did not include any other engagement features. Treatment/therapy/medical websites had scores from 25% to 38% (mean 30%). While all treatment/therapy/medical websites were present on at least one social media platform, none of them had verified accounts. Blog/essay websites had scores from 19% to 50% (mean 35%). News article websites had scores that ranged from 31% to 81% (mean 49%). News article websites typically had verified social media accounts. Additionally, no websites in the treatment/therapy/medical or Blog/essay websites asked users to donate, volunteer, or become members, while two news article websites asked users to become members and one informational website asked users to donate.

### 3.6. Social Media Addiction Content

We evaluated social media addiction content with 22 items (Table 7). All but one website primarily defined social media addiction as using too much social media, and fifteen of the websites mentioned psychological distress and/or impairment in daily functioning as part of the definition (significant distress or impairment is a requirement for all substance use and behavioral addictive disorders listed in the DSM-V). All 23 websites explained symptoms or effects of social media addiction, though the symptoms listed varied: 17 included preoccupation, 10 included mood modification, 11 included tolerance, 17 included withdrawal, 13 included relapse, 18 included relationship problems, 12 included academic problems, 12 included employment problems, and 11 included sleep problems. Only nine of the 23 websites explained that calling disordered social media use an “addiction” is debated, and only five websites explicitly stated that social media addiction is not an official disorder in the DSM-V.

Nine websites explicitly explained how social media addiction is diagnosed, although no formal diagnostic criteria have been published by APA or WHO. Four websites described mental healthcare professionals who can diagnose social media addiction. Three websites mentioned mental healthcare professionals who can treat social media addiction. Seventeen websites described strategies for users to treat social media addiction or reduce social media use. Two websites included materials specifically for healthcare providers, and three websites included materials specifically for family members of an individual with social media addiction.

Overall, the blog/essay websites scored the lowest for social media addiction content, with scores from 9% to 55% (mean 36%) of the 22 items. News article websites scored from 18% to 64% (mean 38%). Treatment/therapy/medical websites had scores that ranged from 18% to 82% (mean 51%). Informational websites had highest number of social media addiction content items, with scores from 27% to 73% (mean 53%). Zero blog/essay websites, one news article website, one treatment/therapy/medical website, and three informational websites explained that social media addiction is not an official disorder according to the DSM-V. Additionally, one blog/essay website, one treatment/therapy/medical website, two news article websites, and five informational websites explicitly mentioned that labeling problematic social media use as an addiction is debatable in the field.

## 4. Discussion

This study aimed to better understand how the most accessed websites for social media addiction present information across multiple metrics. Because of how increasingly common it is for people to use online health information and generally not question the information presented to them [4,29], it was important to conduct a formal, quality assessment of the top returned websites when searching for social media addiction. While the evaluated websites met most of our criteria for design and credibility, results for other metrics varied widely. Social media addiction content and engagement were the metrics that websites scored the lowest in. Low scores among all websites for social media addiction content are particularly concerning because it raises questions about the quality of the content accessed and acted upon by online health information seekers.

Design features were the top-rated metric among all the websites. This is likely due to the ease of designing websites that include these features and well-designed websites engender higher credibility rates from users [30]. Blog/essay websites scored the highest for literacy (excluding the readability measures) while treatment/therapy/medical websites scored the lowest, which may indicate that people with low health literacy may rely on blog/essay websites more because they are easier to read [31]. This was a somewhat unexpected result, given that websites that fall under the treatment/therapy/medical category aim to provide health information to a wide audience. However, previous literature has shown that hospital websites tend to have decreased readability, which can make them difficult for individuals to understand [32,33]. Hence, it is extremely important that healthcare providers spend time with their patients to educate them on the importance of these features in making informed decisions regarding their health. Along with digital literacy, overall literacy is key in making information accessible and actionable to individuals.

The informational category, which had the highest reading level, included websites that can be difficult for the average person to interpret, such as peer-reviewed journal articles and Wikipedia articles. News articles and informational websites generally met the highest number of criteria across the six metrics. News articles scored the highest for credibility, accessibility, and engagement metrics compared to the other classifications. Informational websites scored the highest for design and social media addiction content. Because clear website layout is an important factor for trust formation, it is possible that online health information seekers view informational websites as more credible than other websites [34,35]. However, informational websites also scored the lowest on engagement measures, which may indicate that these websites are somewhat limited in their capacity to spread information. Websites varied widely in their social media presence and verified credibility. However, having a verified social media presence, along with the ability to contact the owner of the website, could influence its overall credibility.

Social media addiction content varied drastically among websites, with these sites providing different definitions, symptoms, and other diagnostic criteria. The symptoms described by these websites primarily fit within Griffiths et al.’s (2014) [7] six component framework, while less than half of the sites listed other aspects, such as sleep problems. We found that many websites, particularly blog/essay and treatment/therapy/medical websites, did not include sources or other means that would lend credibility to their claims about social media addiction. This need for credible information does not seem exclusive to social media addiction, as previous scholarship has cited a similar need for evidence-based information regarding substance abuse disorders, such as cocaine addiction and cannabis addiction [15,16]. Similarly, McKenna et al. (2018) [36] have called for higher involvement from healthcare professionals on food addiction websites. Our findings with social media addiction websites align with these findings, as only three of these websites explicitly stated that their social media addiction content was reviewed by “experts” such as clinicians, healthcare providers, or researchers.

Our findings have implications for laypeople searching for information about social media addiction, as well as healthcare providers. With a lack of guidance from medical bodies, such as APA and WHO, laypeople searching for information about excessive, maladaptive social media use are left to sort through myriad websites covering the topic. As demonstrated above, these websites vary greatly in their quality and content. Therefore, our results might be a guide for healthcare providers in helping individuals make better decisions and judgments about social media addiction information from various internet sources. Additionally, clinicians and therapists should be aware of the variety of information available to their patients online. Healthcare providers can expect that patients who experience difficulty with social media use may have searched the internet for these topics, exposing themselves to the varied information we report here. Our report, therefore, provides clinicians with a survey and analysis of the available online information about social media addiction in the hope that these healthcare providers will better understand their patients when discussing these topics.

As with every study, there are limitations with the current study that deserve mention. Websites can change over time and even disappear. It is possible that the websites we coded between December 2019 and May 2020 will undergo changes in the future. Additionally, search results can change over time as well, depending on the search engine algorithm and the website’s ability to use tools for search engine optimization. These changes over time emphasize the need for ongoing evaluation. Additionally, there may have been instances of website coder error. While the coders were trained and spot-checked by the authors, there could have been an item that was present that the coders believed was absent, and vice-versa. Nevertheless, this study is an important first step in examining presently available, online social media addiction information, which is a topic that is top-of-mind to many people.

Future work may build off the presented results. First, researchers may utilize our methods and replicate our study in the future to evaluate how online information about social media addiction evolves over time. A longitudinal assessment like this will be especially beneficial as criteria for diagnosing social media addiction become more concrete and perhaps formally recognized by clinicians and researchers. Additionally, future work may seek to improve the coding framework used in this study to provide more specific questions about the content of social media addiction websites. Finally, researchers will be able to build hypotheses from our work regarding misinformation about social media addiction and how it spreads through our communities. This misinformation possibly impacts how people seek treatment, as well as how family and friends support individuals who display disordered social media use.

## 5. Conclusions

This analysis serves as an initial exploration of how social media addiction is presented online. As the internet is a widely used tool for seeking health information, it is critically important for healthcare providers and researchers to be aware of the quality of the most accessed websites for social media addiction. In order to remain accessible to a wider audience, websites should maintain a simple layout and use clear language. Perhaps more importantly, a general consensus on a definition for social media addiction, and how it is diagnosed is necessary in order to improve social media addiction-specific content across all website classifications. Because existing websites on social media addiction vary widely in quality across all metrics, it is important to know how to help patients navigate this landscape to help ensure better health outcomes in the future.

## Figures and Tables

**Table 1:** Organization name, category, and URL for assessed websites.

Organization Name	Category	URL	Date Accessed
1. Addiction Center	Treatment/Therapy/Medical	https://www.addictioncenter.com/drugs/social-media-addiction/	27 December 2019
2. Addiction Resource	Treatment/Therapy/Medical	https://addictionresource.com/addiction/technology-addiction/social-media-addiction/	12 May 2020
3. Ashford University	Informational	https://www.ashford.edu/online-degrees/student-lifestyle/causes-of-social-media-addiction-illness	8 May 2020
4. BBC	News Article	https://www.bbc.com/future/article/20180118-how-much-is-too-much-time-on-social-media	12 May 2020
5. Cyberpsychology: Journal of Psychosocial Research on Cyberspace	Informational	https://www.cyberpsychology.eu/article/view/11562/10373	5 Jannuary 2020
6. Forbes	News Article	https://www.forbes.com/sites/alicegwalton/2017/06/30/a-run-down-of-social-medias-effects-on-our-mental-health/#7ea631222e5a	12 May 2020
7. Huffington Post	News Article	https://www.huffpost.com/entry/biological-psychological-reasons-for-social-media_b_58c279a7e4b0c3276fb78388	12 May 2020
8. Journal of Addiction Research & Therapy	Informational	https://www.omicsonline.org/social-networking-addiction-emerging-themes-and-issues-2155-6105.1000e118.php?aid=22152	13 May 2020
9. Lifewire	Blog/Essay	https://www.lifewire.com/what-is-social-networking-addiction-2655246	11 May 2020
10. Mediakix	Blog/Essay	https://mediakix.com/blog/social-media-addiction-statistics/	11 May 2020
11. NewLifeOutlook: Addiction	Informational	https://addiction.newlifeoutlook.com/social-media-addiction/?all=1	10 May 2020
12. Northpoint Recovery	Treatment/Therapy/Medical	https://www.northpointwashington.com/process-addiction/social-media-addiction.php	12 May 2020
13. Paradigm Treatment	Treatment/Therapy/Medical	https://paradigmmalibu.com/teen-social-media-addiction-treatment/	12 May 2020
14. Perspectives of Troy	Treatment/Therapy/Medical	https://perspectivesoftroy.com/social-media-addiction/	12 May 2020
15. Psychology Today	Blog/Essay	https://www.psychologytoday.com/us/blog/in-excess/201805/addicted-social-media	30 March 2020
16. Psycom	Informational	https://www.psycom.net/iadcriteria.html	12 May 2020
17. Social Champ	Blog/Essay	https://blog.socialchamp.io/social-media-addiction-statistics/	11 May 2020
18. South University	Informational	https://www.southuniversity.edu/whoweare/newsroom/blog/does-social-media-addiction-really-exist-31795	11 May 2020
19. Sovereign Health	Treatment/Therapy/Medical	https://www.sovteens.com/treatment-programs/teen-behavioral-health/social-media-addiction/	16 April 2020
20. The Washington Post	News Article	https://www.washingtonpost.com/news/theworldpost/wp/2018/04/25/social-media-addiction/	30 December 2019
21. The Wisdom Post	Blog/Essay	https://www.thewisdompost.com/essay/addiction/social-media-addiction/social-media-addiction-meaning-symptoms-causes-effects-treatment/1293	3 Jannuary 2020
22. Wikipedia	Informational	https://en.wikipedia.org/wiki/Problematic_social_media_use	9 May 2020
23. Your English Success Today	Blog/Essay	https://www.fishsuccesstoday.com/english-language-blog/social-media-addiction-causes-effects-and-possible-solutions	11 May 2020

**Table 2:** Design assessment.

Item	Yes (*n* ^a^)	No (*n* ^a^)
Can users access the website correctly on Internet Explorer?	23 ^1–23^	0
Can users access the website correctly on Firefox?	23 ^1–23^	0
Can users access the website correctly on Chrome?	23 ^1–23^	0
Can users access the website correctly on Safari?	23 ^1–23^	0
Can users access the website correctly using a mobile phone?	23 ^1–23^	0
Is there a paywall to read the full article/page?	0	23 ^1–23^
Are logos consistent throughout the website?	23 ^1–23^	0
Are page headers consistent throughout the website?	23 ^1–23^	0
Are graphics consistent throughout the website?	23 ^1–23^	0
Are colors consistent throughout the website?	23 ^1–23^	1 ^23^
Is font size consistent throughout the website?	23 ^1–23^	0
Is there a consistent use of style throughout the website?	23 ^1–23^	0
Does the website use the proper capitalization of sentences?	23 ^1–23^	0
Are items correctly aligned on the page?	23 ^1–23^	0
Does the website provide a search function?	19 ^1–9,11,14–22^	4 ^1^^0,12,13,23^
If the website provides a search function, is the scope of the ^search indicated?	7 ^1,3,7,11,15,21,22^	12 ^2,4–6,8,9,14,16–20^
Are the search results displayed clearly?	19 ^1–9,11,14–22^	0
Is there a home page link on all pages in the website?	23 ^1–23^	0
Is there a Help section?	3 ^20,22,23^	20 ^1–19,21^
Is the most important information on the top of the page? “Above the fold” (No scrolling necessary).	7 ^1,5,12,14,15,18,22^	12 ^2–4,6–11,13,16,17,19–21,23^
Is the home page simple? (White space used, no clutter, etc.)	23 ^1–23^	0
Does the top window bar display the article name?	23 ^1–23^	0
Is there a logo in the top window bar that matches the website?	22 ^1–12,14–23^	1 ^13^
Are “crumb trails” used to help users understand where they are in the website?	8 ^2,3,5,9,12,13,16,19^	15 ^1,4,6–8,10,11,14,15,17,18,20–23^
Is there a site map?	18 ^1–3,7–10,12–14,16–23^	5 ^4–6,11,15^

^a^ See Table 1 for the websites that correspond to the superscript numbers.

**Table 3:** Credibility assessment.

Item	Yes (*n* ^a^)	No (*n* ^a^)
Does the website note an author, editor, and/or reviewer?	14 ^1,3–9,11,15–17,20,21^	9 ^2,10,12–14,18,19,22,23^
Does the website explicitly disclose its purpose/goal/mission?	15 ^2–4,7,9–12,17–23^	8 ^1,5,6,8,13–16^
If the purpose/goal/mission is not explicitly disclosed, is information given about the organization’s purpose?	7 ^1,5,6,8,13–15^	1 ^16^
Does the website indicate if it is copyrighted?	20 ^1–9,11–20,23^	3 ^10,21,22^
Is the copyright year “2020”?	19 ^1,2,4–13,15–20,23^	4 ^3,14,21,22^
Does the website have a “terms of use” or “terms of service” section?	15 ^2–4,6,7,9,11,14–16,18–22^	8 ^1–5,8,10,12,13,17,23^
Is a privacy statement made available to users?	18 ^1–4,6,7,9–11,13–16,18–22^	5 ^5,8,12,17,23^
Within the privacy disclaimer, is there a mention of cookies?	18 ^1–7,9–11,13–16,18,19,21,22^	5 ^8,12,17,20,23^
Does the website present reference for their informational content at least once?	12 ^2–5,8,10,11,15,18,20–22^	11 ^1,6,7,9,12–14,16,17,19,23^
Is the creation date of the website’s content on display?	14 ^2–9,11,15,17,18,20,23^	9 ^1,10,12–14,16,19,21,22^
Is the date of the last update displayed?	7 ^1,2,5,7,11,16,22^	16 ^3,4,6,8–10,12–15,17–21,23^
Was the content reviewed by “experts”?	3 ^1,5,8^	20 ^2–4,6,7,9–23^

^a^ See Table 1 for the websites that correspond to the superscript numbers.

**Table 4:** Accessibility assessment.

Item	Yes (*n* ^a^)	No (*n* ^a^)
Can users adjust the font size?	0	23 ^1–23^
If the font can be adjusted, are the pages usable when the text is enlarged?	0	23 ^1–23^
Do the links look clickable?	10 ^1–18,20,22^	3 ^19,21,23^
Are written out webpages clickable?	3 ^3,5,22^	2 ^21,23^
Does the spacing of content change when users make the window smaller/bigger?	22 ^1–4,6–23^	1 ^5^
Are users able to use the keyboard to navigate the webpage?	23 ^1–23^	0
Does the website use a focus indicator to show where the user is on the webpage?	17 ^1–4,6–8,11–13,15,16,18–20,22,23^	6 ^5,9,10,14,17,21^
Is there sufficient color contrast?	23 ^1–23^	0
Is there an option on the website that will allow users to view the exact webpage in a different language?	3 ^8,13,22^	20 ^1–7,9–12,14–21,23^
Is there an option on the website that will allow users to listen to written material?	0	23 ^1–23^
Is there flashing or blinking on the page (not including advertisements)?	1 ^17^	22 ^1–16,18–23^
Are there videos on the webpage?	4 ^4,7,13,14^	19 ^1–3,5,6,8–12,15–23^
If there are videos, are the videos captioned?	1 ^4^	3 ^7,13,14^
Are pictures used on the website (not including advertisements)?	20 ^1–4,6,7,9–14,16–23^	3 ^5,8,15^
If there are pictures, is there text that explains images on the website?	4 ^4,7,13,20^	16 ^1–3,6,9–12,14,16–19,21–23^

^a^ See Table 1 for the websites that correspond to the superscript numbers.

**Table 5:** Literacy assessment.

Item	Yes (*n* ^a^)	No (*n* ^a^)
Are lists used to break up blocks of text?	23^1–23^	0
Do the websites provide a “hover-over” or clickable link to definitions for medical terms?	14 ^1,2,6,7,9–12,14–16,18,20,22^	9 ^3–5,8,13,17,19,21,23^
Are numbers used?	22 ^1–13,15–23^	1 ^14^
Are fractions used?	7 ^1,4,9,10,12,13,17^	16 ^2,3,5–8,11,14–16,18–23^
Are percentages used?	15 ^1–5,7,8,10–13,16,17,21,22^	8 ^6,9,14,15,18–20,23^
Are decimals used?	9 ^1,5,10–13,16,17,21^	14 ^2–4,6–9,14,15,18–20,22,23^
Are ranges used?	11 ^1,3,5,7,9–11,17,18,21,22^	12 ^2,4,6,8,12–16,19,20,23^
Are descriptive statistical terms used (e.g., mean, average, median, etc.)?	7 ^5,7,10–12,17,21^	16 ^1–4,6,8,9,13–16,18–20,22,23^
Are temporal terms used (e.g., minutes, hours, days, years, etc.)?	22 ^1–18,20–23^	1 ^19^
Are graphs, charts, or tables used?	2 ^4,5^	21 ^1–3,6–23^
Can users easily print information on the website?	18 ^1,3,5–8,10–14,16–19,21–23^	5 ^2,4,9,15,20^
Can users easily send information to someone else?	10 ^3,4,7,9–11,15,18,20,23^	13 ^1,2,5,6,8,12–14,16,17,19,21,22^
Are there advertisements used on the website?	9 ^4,6,7,9–11,15,16,20^	14 ^1–3,5,8,12–14,7–19,21–23^
If yes, are they labeled as advertisements?	7 ^4,6,7,9,11,15,20^	16 ^1–3,5,8,10,12–14,16–19,21–23^
Do the advertisements move, flash, change, etc.?	5 ^6,9,10,15,20^	18 ^1–5,7,8,11–14,16–19,21–23^

^a^ See Table 1 for the websites that correspond to the superscript numbers.

**Table 6:** Engagement assessment.

Item	Yes (*n* ^a^)	No (*n* ^a^)
Is there a “Contact Us” section?	20 ^1–14,16,19–23^	3 ^15,17,18^
Does the website have a presence on Facebook?	20 ^1–4,6–15,17–21,23^	3 ^5,16,22^
If there is a presence on Facebook, is it a verified account?	8 ^3,4,6,7,9,15,18,20^	12 ^1,2,8,10–14,17,19,21,23^
Does the website have a presence on Twitter?	14 ^1–4,8,10–12,14,15,17–20^	9 ^5–7,9,13,16,21–23^
If there is a presence on Twitter, is it a verified account?	4 ^3,4,15,20^	10 ^1,2,8,10–12,14,17–19^
Does the website have a YouTube channel?	10 ^3,8,12,13,15,17–20,23^	13 ^1,2,4–7,9–11,14,16,21,22^
If there is a YouTube channel, is it a verified account?	2 ^18,20^	8 ^3,8,12,13,15,17,19,23^
Does the website have a presence on Pinterest?	7 ^3,13–15,17,19,20^	16 ^1,2,4–12,16,18,21–23^
If there is a presence on Pinterest, is it a verified account?	1 ^20^	6 ^3,13–15,17,19^
Does the website have a presence on Instagram?	11 ^1–3,10,12,13,15,17,18,20,23^	12 ^4–9,11,14,16,19,21,22^
If there is a presence on Instagram, is it a verified account?	2 ^3,20^	9 ^1,2,10,12,13,15,17,18,23^
Can an individual easily post/share content from the website to their social media profile?	13 ^2–4,6–11,15,18,20,21^	10 ^1,5,12–14,16,17,19,22,23^
Can an individual receive email updates?	10 ^3,4,6,7,9,10,11,17,20,23^	13 ^1,2,5,8,12–16,18,19,21,22^
Does the website ask individuals to donate?	1 ^22^	22 ^1–21,23^
Does the website ask individuals to become a member/actively involved?	2 ^7,20^	21 ^1–6,8–19,21–23^
Does the website ask individuals to volunteer?	0	23 ^1–23^

^a^ See Table 1 for the websites that correspond to the superscript numbers.

**Table 7:** Social media addiction content assessment.

Item	Yes (*n* ^a^)	No (*n* ^a^)
Does the website primarily define social media addiction as using too much social media?	22 ^1–11,12–23^	1 ^10^
Does the website define social media addiction as using too much social media so that psychological distress occurs and/or daily functioning is impaired?	15 ^1,2,4,5,8,9,11,13,15–17,20–22^	8 ^3,6,7,10,12,14,18,19,23^
Does the website explain what the symptoms/effects of social media addiction are?	23 ^1–23^	0
Does the website describe the following as symptoms/effects of social media addiction?		
Preoccupation?	17 ^1,3,5–8,11–13,15–17,19–23^	6 ^2,4,9,10,14,18^
Mood modification?	10 ^1,4–6,8,16,17,20–22^	13 ^2,3,7,9–15,18,19,23^
Tolerance?	11 ^1,3,6,8,12,13,15,16,19,20,22^	12 ^2,4,5,7,9–11,14,17,18,21,23^
Withdrawal?	17 ^1–3,5–8,11–14,16,17,19–22^	6 ^4,9,10,15,18,23^
Relapse?	13 ^1,3–6,8,12,15–17,19,20,22^	10 ^2,7,9–11,13,14,18,21,23^
Interpersonal relationship problems (e.g., ruining relationships, divorce)?	18 ^1,3–6,8,9,11,13,15–23^	5 ^2,7,10,12,14^
Academic problems (e.g., dropping out, failing)?	12 ^1,2,5,8,9,11,13,15,16,19,20,23^	11 ^3,4,6,7,10,12,14,17,18,21,22^
Employment problems (e.g., job loss, poor job performance)?	12 ^1,2,8,9,11,15–21^	11 ^3,4–7,10,12–14,22,23^
Sleep problems (e.g., staying up too late, not feeling rested when waking)?	11 ^2,5,9–11,13,16,17,19,22,23^	12 ^1,3,4,6–8,12,14,15,18,20,21^
Does the website explain that calling problematic use of social media an “addiction” is debatable?	9 ^4,7–9,11,12,16,18,22^	14 ^1–3,4,6,10,13–15,17,19–21,23^
Does the website explain that social media addiction is not an official disorder (e.g., described in the DSM-V)?	5 ^4,11,16,19,22^	18 ^1–3,5–10,12–15,17,18,20,21,23^
Does the website explicitly tell users about how social media addiction is diagnosed?	9 ^1,3,12,15–17,19,20,22^	14 ^2,4–11,13,14,18,21,23^
Does the website describe mental healthcare professionals who can diagnose social media addiction?	3 ^4,15,20^	20 ^1–3,5–14,16–19,21–23^
Does the website describe mental healthcare professionals who can treat social media addiction?	4 ^1,13,19,20^	19 ^2–12,14,15–18,21–23^
Does the website tell users about strategies to treat an addiction/reduce social media use?	17 ^1,2,4,5,11–23^	6 ^3,6–10^
Does the website have first person accounts about living with social media addiction?	1 ^18^	22 ^1–17,19–23^
Does the website have materials specifically for healthcare providers?	2 ^12,14^	21 ^1–11,13,15–23^
Does the website explicitly have materials for family members of an individual with social media addiction?	3 ^2,13,15^	20 ^1,3–12,14,16–23^
Does the website talk about health insurance coverage for social media addiction?	3 ^2,13,19^	20 ^1,3–12,14–18,20–23^

^a^ See Table 1 for the websites that correspond to the superscript numbers.

## Data Availability

Not applicable.

## Appendix A

Search terms for social media addiction

1. Social media addiction
2. Dependence on social media
3. Problematic social media use
4. Excessive social media use
5. Social media addiction effects
6. Social media addiction facts
7. Social media addiction causes
8. Social media addiction solutions
9. Social media addiction treatment
10. Social media addiction help
11. Social media addiction symptoms
12. Social media addiction statistics
13. Social media addiction study
14. Social media addiction research
15. Social media addiction signs
16. Social media addiction definition
17. Social media addiction types
18. Social media addiction evidence
19. Effects of social media addiction
20. Facts of social media addiction
21. Causes of social media addiction
22. Solutions for social media addiction
23. Treatment for social media addiction
24. Help for social media addiction
25. Symptoms of social media addiction
26. Statistics of social media addiction
27. Study of social media addiction
28. Research on social media addiction
29. Signs of social media addiction
30. Definition of social media addiction
31. Types of social media addiction
32. Evidence of social media addiction
33. Why are people addicted to social media
34. Is social media addiction a disorder
35. How much social media use is too much
36. How much social media use is an addiction
37. How much social media use is a problem
38. How much social media use is excessive
